# Euler’s Elastica-Based Biomechanics of the Papillary Muscle Approximation in Ischemic Mitral Valve Regurgitation: A Simple 2D Analytical Model

**DOI:** 10.3390/ma12091518

**Published:** 2019-05-09

**Authors:** Francesco Nappi, Angelo Rosario Carotenuto, Sanjeet Singh Avtaar Singh, Christos Mihos, Massimiliano Fraldi

**Affiliations:** 1 Centre Cardiologique du Nord de Saint-Denis, Paris 36 Rue des Moulins Gmeaux, 93200 Saint-Denis, France; 2 Department of Structures for Engineering and Architecture, University of Napoli Federico II, 80125 Naples, Italy; angelorosario.carotenuto@unina.it (A.R.C.); fraldi@unina.it (M.F.); 3 Department of Cardiac Surgery, Golden Jubilee National Hospital, Clydebank G81 4DY, UK; sanjeetsinghtoor@gmail.com; 4 Columbia University Division of Cardiology at the Mount Sinai Heart Institute, Miami Beach, FL 33140, USA; drcmihos@gmail.com

**Keywords:** mitral valve, papillary muscle approximation, biomechanics

## Abstract

Ischemic mitral regurgitation (IMR) occurs as an adverse consequence of left ventricle remodeling post-myocardial infarction. A change in mitral valve configuration with an imbalance between closing and tethering forces underlie this pathological condition. These abnormalities lead to impaired leaflet coaptation and a variable degree of mitral regurgitation, which can in turn influence the ventricular filling status, the heart rhythm and the afterload regardless of the residual ischemic insult. The IMR correction can be pursued through under-sizing mitral annuloplasty and papillary muscle approximation to restore the mitral valve and left ventricle physiological geometry to, consequently, achieve normalization of the engaged physical forces. Because the structures involved undergo extremely large deformations, a biomechanics model based on the Euler’s Elastica –the mitral leaflet– interlaced with nonlinear chordae tendineae anchored on papillary muscles has been constructed to elucidate the interactions between closing and tethering forces. The model takes into account the actual updated geometrical and mechanical features of the valvular and subvalvular apparatuses in physiological and IMR conditions, as well as in case of papillary muscle approximation, finally furnishing ad hoc geometry-based mathematical relations that could be utilised to support—and optimize—the relevant choices in cardiac surgery.

## 1. Introduction

Ischemic mitral regurgitation (IMR) is an acquired geometric dysfunction of mitral valve motion secondary to myocardial infarction that is characterized by post-ischemic adverse left ventricular remodelling, left ventricular distortion and, finally, enlargement of left side cardiac chambers. The main pathophysiological mechanism behind the IMR is the displacement of papillary muscles along a posterior, apical or lateral vector. Several biomechanical features are involved in determining an imbalance between tethering and closing forces at level of valvular and subvalvular mitral valve apparatus. Tethering forces are identified in papillary muscles displacement, annular dilatation, left ventricle (LV) dilatation and LV sphericity while closing forces are composed by altered mitral systolic annular contraction, reduction of LV contractility, global LV dyssynchrony and papillary muscle dyssynchrony. Patients with moderate to severe IMR are destined for surgical treatment and the American College of Cardiology/American Heart Association (ACC/AHA) guidelines recommend consideration of mitral-valve repair or chordal-sparing replacement [1]. However, guidelines do not indicate which is superior between the two approaches. Expert consensus favours the surgical correction of the mitral valve abnormalities addressing both the annulus (which is normally distorted but not always dilated) and the subvalvular apparatus [2,3]. Randomized controlled trial and several observational studies revealing that mitral valve repair with restrictive annuloplasty (RA) has been associated with a high rate of recurrent mitral regurgitation and need for repeat surgery [4,5,6]. MR recurrence is due to augmented leaflet tethering caused by the anterior displacement of the posterior leaflet [3,4,5,6,7]. We pioneered the surgical treatment of the subvalvular apparatus as a key to restore mitral valve function in moderate and severe IMR [8]. In our randomized controlled clinical trial, designed to compare 96 patients who underwent either combined papillary muscle approximation (PMA) and restrictive mitral annuloplasty or undersized valve annuloplasty alone, we observed that the relative difference in the geometrical profiles of the mitral valve in IMR was at the basis of the non-homogenous results [5]. Mitral-valve repair with a restrictive annuloplasty, in both symmetric and asymmetric tethering, can result in a still abnormal interpapillary distance (IPD), augmented leaflets tethering, due to the anterior and posterior displacement of the leaflets, and persistent increase of tenting area, anticipating the persistence or recurrence of mitral regurgitation. Addition of papillary muscle surgery using expanded Polytetrafluoroethylene (E-PTFE) for the rapprochement produced a significant benefit as reducing the IPD and positively contributing to mitral and LV geometry [4]. However, in patients with symmetric tethering and significantly dilated LV chambers were still harnessed by a percentage of mitral regurgitation recurrence notwithstanding the achievement of an IPD reduction of 25%–30% [4,6]. Persistent tethering in over-sized ventricular chambers complicates the long-term results of surgery. Therefore, these biomechanical consequences need to be further investigated in order to elucidate the mechanism underlying failure of mitral repair. This study aims to develop a biomechanical model able of tailoring the type and extent of the subvalvular surgical approach in each case, also encouraged by recent successful biomechanically-driven developments in vascular surgery [9]. An analytical model is used to obtain measures of the geometric variables reflecting the parameters normally used to characterize the type and degree of IMR (anteroposterior diameter of the mitral annulus, tenting height associated with tenting area, postoperative interpapillary distance, and papillary muscle displacement resulting from their approximation). We believe that the results of biomechanical analysis here addressed can give valuable information to health providers, cardiologists and surgeons, for IMR diagnosis and treatment.

**Figure 1:**
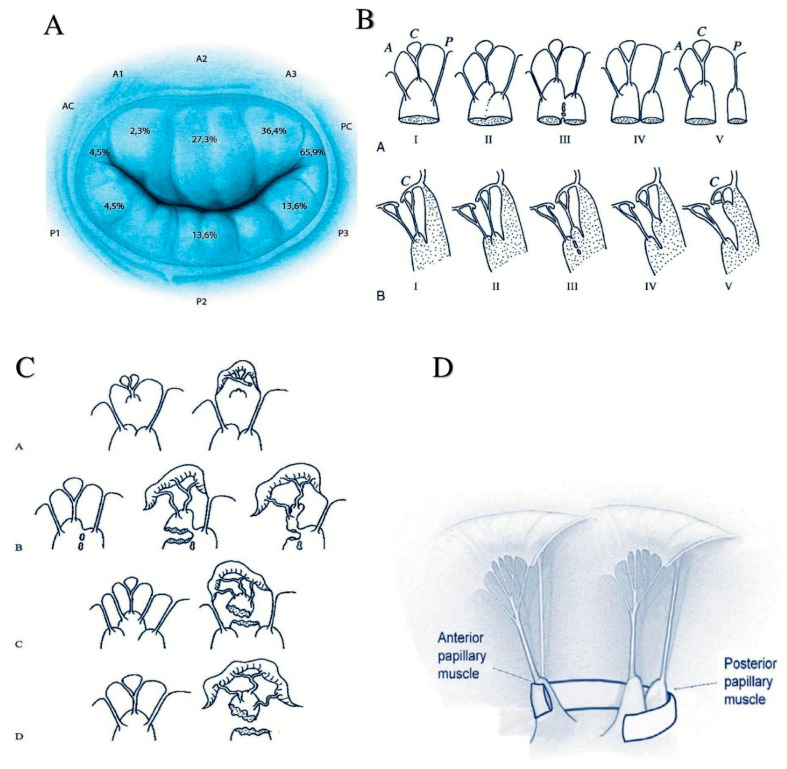
(**A**). Distribution and incidence of valve annular stress after ischemic mitral regurgitation IMR. Valvular region is organized in anterior and posterior leaflet. Each leaflet is divided in three segments or scallops that are named A1, A2, A3 and P1, P2, P3 relating to anterior or posterior cusp, respectively. The leaflets are attached to fibrous circular annulus and join in the anterior and posterior commissure, respectively, giving to the mitral valve the appearance of a curtain that is closed during systole. Tethering greatly affects the A3 and P3 segments and the posterior commissure (PC); (**B**) Segmentation and morphological types of papillary muscles; I, single uniform unit; II, groove with two apexes; III, fenestrations with muscular bridges; IV, complete separation in two adjacent heads; V, complete separation with two distant heads. Division can occur according to two directions: (A small) Division in a sagittal plane leading to a separate posterior leaflet head. (B small) Division in a coronal plane leading to a separate commissural head. A, anterior leaflet; C, commissure; P, posterior leaflet; (**C**) Mechanisms of ischemic mitral valve regurgitation for chordae stress and stretch with partial or total necrosis of papillary muscle approximation (PMA): (A small) Necrosis of a separate commissural head (inserted close to the annulus) with rupture of the anchorage of the commissural chord. (B small) Necrosis of a single head papillary muscle subdivided in multiple heads with partial rupture. (C small) Necrosis of a fenestrated papillary muscle with detachment of its main insertion: “incomplete” rupture. With time, incomplete rupture mimics papillary muscle elongation. (D small) Single papillary muscle with complete and total rupture; (**D**) Papillary muscle approximation surgery using 4 mm tube of expanded politetrafluoroethilene to encercle the body of papillary muscle(s) (PMs). Posterior papillary muscle is anatomically of type III-V.

**Figure 2:**
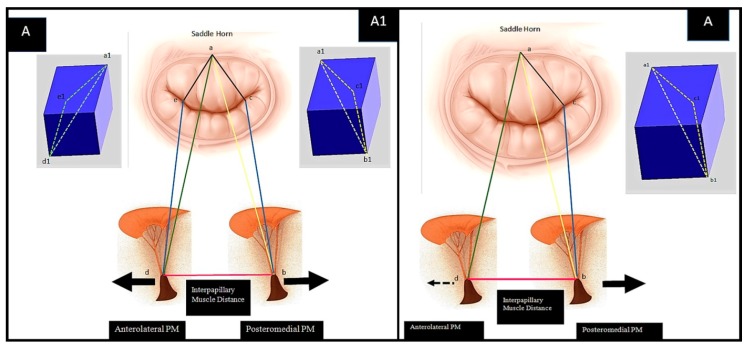
Schematic representation of symmetric and asymmetric pattern of mitral valve tethering. (**Left**) Symmetric tethering pattern. With apical and lateral vector components, the posteromedial papillary muscle and both mitral leaflets are medially displaced, resulting in symmetric leaflet tethering. Of note, primarily apical displacement of both PMs favours less leaflet tethering, while a more medial shift results in increased tethering of both leaflets. (a–b) Posterior PM tethering, distance between the posteromedial PM tip and the saddle horn; (a–d) anterolateral PM tethering, distance between the anterolateral PM tip and the saddle horn; (a–c) anterior component of posterior PM tethering; (b–c) posterior (inferior) component of posteromedial PM tethering; (a–d) anterolateral PM tethering with its components (a–e) and (e–d). (**Right**) Asymmetric tethering pattern. A posterior vector imparts a posterior (major) and apical (minor) displacement of the postero medial papillary muscle (PMPM) resulting in asymmetric tethering. (a–b) Posterior PM tethering, distance between the posteromedial PM tip and the saddle horn; (a–d) anterolateral PM tethering, distance between the anterolateral PM tip and the saddle horn; (a–c) anterior component of posterior PM tethering; (b–c) posterior (inferior) component of posteromedial PM tethering; (a–d) anterolateral PM tethering is less implicated and characterized by apical and posterolateral components. A and A1—tetrahedron representation of symmetric tethering pattern. A—tetrahedron representation of posteromedial PM symmetric tethering. Symmetric anterior and posterior leaflet tethering. A + A1—tetrahedron representation both of PMs.

Figure 1 shows the anatomy of valvular and subvalvular mitral valve apparatus in normal conditions and after IMR correction (Figure 1; Panel A–D). Patients who develop IMR undergo anatomical changes of the entire valve, for which the decisive factor for biomechanical implications is tethering. The biomechanical distortion of the LV chamber undoubtably compromises the overall equilibrium between the tethering and closing forces. The subvalvular chordae tendineae function, LV contractility, alongside the natural bending of the mitral valve and tissue in situ pre-stressing, to ensuring a physiologically healthy configuration is inevitably lost. IMR presents with two types of tethering shapes, symmetric and asymmetric, as schematically represented in Figure 2. In the symmetric form, the left ventricular chamber is significantly dilated and papillary muscles are displaced along an apical or lateral vector. The PMs normally anchor chordae for both leaflets; therefore, due to their migration, traction is exerted on both the anterior and posterior leaflets causing impaired cusp coaptation and restriction. The normal anterior point of coaptation, which indicates the correct orientation of the valve, migrates posteriorly. The flux of regurgitation through mitral valve (i.e., the regurgitant jet) has a central direction due to the symmetric geometrical disturbance and the homogeneous traction on the leaflets. The asymmetric tethering shape is characterized by the posterior migration of the postero medial papillary muscle (PMPM) generally involved in inferior myocardial infarctions. Left ventricular distortion is more pronounced with a marked increase of diastolic sphericity index, reduced ventricular dilatation and higher degree of beta angle tethering. The anterior coaptation point migrates posteriorly, parallel to the displacement vector, and the posterior leaflet tenting is more pronounced resulting in asymmetric direction of the regurgitant jet.

**Figure 3:**
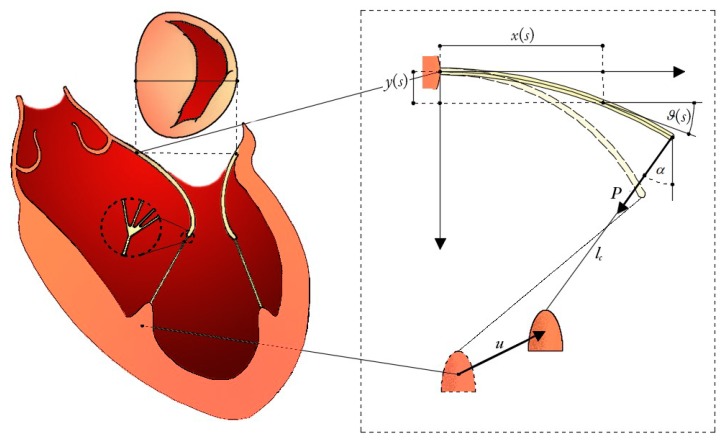
Hand-made sketch of the in-plane mechanical modelling of the mitral valve nonlinear bending combined with tethering due to the interaction with hyperelastic chordae anchored to the moving PM site.

Papillary muscle approximation (PMA) combined with restrictive annuloplasty (RA) has proved beneficial in reducing augmented leaflet tethering for both symmetric and asymmetric shapes, as illustrated in Figure 1, Panel D [4,5,6,8]. From a biomechanical standpoint, the mechanics of the heart valve has usually been treated by means of finite element models [10,11]. Despite the accuracy of results furnished by such mechanical analyses in terms of both 3D geometric reconstructions and stress states, the numerical outcomes were somehow patient-specific, while the mechanical exchange of forces between valve leaflets and subvalvular chordae tendineae were not investigated thoroughly, as was the role played by the relevant geometrical and kinematical variables, measurable via commonly used techniques and recognized as indicators of IMR status in the PMA. To gain insights into the prediction of the effectiveness of the postoperative outcomes from the knowledge of the preoperative configuration in the PMA for IMR treatment, a biomechanical model engaging all the aforementioned clinically-measurable and surgically-significant data has been built up by coupling Euler’s Elastica theory [12,13,14] with nonlinear and moving boundary conditions that faithfully describe the behaviour of the valve-chordae system. In particular, the proposed biomechanical scheme made of two nonlinear cantilever beams—the mitral valve leaflets—anchored at their tips to hyperelastic elements—representing the chordae tendineae—that transmit the tethering forces to the papillary muscles (PM) (see Figure 3). This allows the model to inform us on how the large deflection of the leaflets is associated to the chordae tethering forces and how, in turn, they are transferred to the PM loci as a function of their moving positions during PMA. In presenting the theory, we first separately describe the kinematics and the mechanical behaviour of each structural element of the system, then coupling them through equilibrium and compatibility equations. After that, the proposed mathematical strategy is applied to guide the undersizing restrictive annuloplasty and to optimize papillary muscle approximation.

## 2. Materials and Methods

### 2.1. Mitral Leaflets Modelled as Euler’s Elastica

We considered a planar problem, in which the flexural response and the deformed shape of the mitral valve leaflets were modelled as nonlinear cantilever beams subjected to end-point loads, whose directions and intensities accord the axial tensile forces in the chorda tendinea, guaranteeing geometric compatibility and equilibrium. Experimental observations have recently shown that the flexural response of aortic valves can be depicted in a satisfactorily manner by means of a beam bending test [15] Here, although no 3D characterization occurred, this feature was preserved and extrapolated to model the in-plane-projected kinematics of the mitral valve (MV), by adopting a fully analytical and nonlinear approach. Therefore, the well-known solutions of an inextensible cantilever Elastica with length l and subjected to vertical end-point load [12,16] were modified in order to accommodate the action of an arbitrary oriented force, whose direction was guided by the chorda connecting the leaflet free-edge to the respective PM, as illustrated in Figure 3. Under these conditions, the parametric shape of the deformed elastica reads: (details of the standard mathematical formulation for the nonlinear Elastica can be found in well-established literature [12,16]):

(1) $$\begin{array}{l} \frac{x\left(s\right)}{l}=\frac{2k\cos\alpha }{\omega }\left(\cos\varphi_{\alpha }-\cos\varphi \left(s\right)\right)-\frac{\sin\alpha }{\omega }(F\left[\varphi \left(s\right),k\right]-2E\left[\varphi \left(s\right),k\right]+2E\left[\varphi_{\alpha },k\right]-F\left[\varphi_{\alpha },k\right])\\ \frac{y\left(s\right)}{l}=\frac{\cos\alpha }{\omega }\left(F\left[\varphi \left(s\right),k\right]-2E\left[\varphi \left(s\right),k\right]+2E\left[\varphi_{\alpha },k\right]-F\left[\varphi_{\alpha },k\right]\right)+\frac{2k\sin\alpha }{\omega }\left(\cos\varphi_{\alpha }-\cos\varphi \left(s\right)\right) \end{array}$$

where s∈[0,1] is the dimensionless curvilinear abscissa (Figure 3), while the function and the eccentricity k are given by:

(2) $$\varphi \left(s\right)=\mathrm{am}\left[\omega \;s+F\left[\varphi_{\alpha },k\right],\;k\right],$$

(3) $$2k^{2}=1+\sin\left(\theta_{l}-\alpha \right),$$

(4) $$2k^{2}\sin^{2}\varphi_{\alpha }=1-\sin\alpha$$

Relations (1–5) involve the use of the implicit incomplete functions am [·], F [·] and E [·], which denote, respectively, the Jacobi amplitude function and the elliptic integral of the first kind with elliptic modulus $k^{2}$. The other parameters appearing in the Equations (1–5) are the load inclination α (w.r.t. the *y*-axis), the end point angle *θ_l_* (formed with the horizontal axis) and the coefficient *ω*^2^ = *P l*^2^*/B*, *B* being the bending stiffness and the load *P* obeying the equation:

(5) $$P=\frac{B}{l^{2}}{\left(K\left[k\right]-F\left[\varphi_{\alpha },k\right]\right)}^{2}$$

Equations (1) and (2), evaluated in s = 1, allow to calculate the beam free-edge position $b=\left\{x_{l},y_{l}\right\}$:

(6) $$x_{l}=\frac{2k\cos\alpha l}{\omega }\cos\varphi_{\alpha }-\frac{\sin\alpha l}{\omega }\left(K\left[k\right]-2E\left[k\right]+2E\left[\varphi_{\alpha },k\right]-F\left[\varphi_{\alpha },k\right]\right)$$

(7) $$y_{l}=\frac{\cos\alpha l}{\omega }\left(K\left[k\right]-2E\left[k\right]+2E\left[\varphi_{\alpha },k\right]-F\left[\varphi_{\alpha },k\right]\right)+\frac{2k\sin\alpha l}{\omega }\cos\varphi_{\alpha }\psi_{c}$$

The shape of each valve leaflet is then governed by the Lagrangian parameters *θ_l_* and *α*, determined by the slope of the chorda tendinea. Thus, the *α* results itself a function of both the angle *θ_l_* and the position of the papillary muscle, in the problem at hand, was subjected to a displacement vector u to simulate the PM approximation.

### 2.2. Hyperelasticity of the Chordae Tendinee

Chordae experience uniaxial tension in physiological conditions and are modelled here as one dimensional (incompressible) elements exhibiting a Fung-type behaviour, with strain energy density:

(8) $$\psi_{c}\left(\varepsilon \right)=\frac{c_{1}}{c_{2}}\left(e^{c_{2}\varepsilon }-1\right)-c_{1}\varepsilon$$

where *ε* = (*λ*^2^−1)/2 is the Green strain, the stretch *λ = l_c_/L_c_* being the ratio between current and reference chorda lengths. In our problem, the postoperative unknown length *l_c_* will explicitly depend upon both the angle *θ_l_* and the PM displacement u, while the unknown reference length *L_c_* is evaluated by means of a pull-back operation, starting from the observed pre-operative chorda. Utilizing material constants *c*_1_ and *c*_2_ from literature stress–strain tests [17], the tensile Cauchy stress σ is calculated as:

(9) $$\sigma =\frac{\partial \psi_{c}}{\partial \varepsilon }=c_{1}\left(e^{c_{2}\varepsilon }-1\right)=T\lambda$$

where *T = P/A_ref_* denotes the nominal stress (i.e., the edge load per unit reference cross-sectional area of the tendon).

### 2.3. Chorda-Leaflet Coupling and Overall System Interaction

The biomechanical modelling of restrictive annuloplasty combined with PMA is conceptually sketched in Figure 3. Two leaflets—namely the anterior and the posterior—are oppositely placed at a distance equal to the MV diameter. Two tethering chordae connect the free edges of the leaflets with the respective PMs (i.e., the antero-lateral and the postero-medial PM), located at the so-called inter-papillary muscle distance (IPD). The clinical determination of some geometric parameters of interest, which are known to discriminate the IMR grade in surgical practice [18,19,20], allows characterization of the preoperative structural configuration to successively obtain the reference parameters needed for the prediction of postoperative outcomes. With reference to the biomechanical model, the parameters required for describing the shape of the structures at each configuration are: the free edge slopes of the two leaflets, say $\theta_{l}{}^{a}$ and $\theta_{l}{}^{p}$, the superscripts *a* and *p,* respectively, indicating the components belonging to the anterior and the posterior structures, and the chordae angles *α^a^* and *α^p^*. The equilibrium at each leaflet free edge-chorda connection node requires:

(10) $$\sigma_{k}^{i}=\lambda_{k}^{i}\frac{P_{k}}{A_{ref}}$$

where *i* = {*a,p*} and the additional subscript *k* denotes the configuration at which equilibrium is needed. Furthermore, to simulate the RA and PMA, additional unknowns are introduced (i.e., the mitral annulus restriction *r* and the PM displacement vectors **u**^a^ and **u**^p^) (Figure 3).

#### The Pre-Operative Configuration

**Figure 4:**
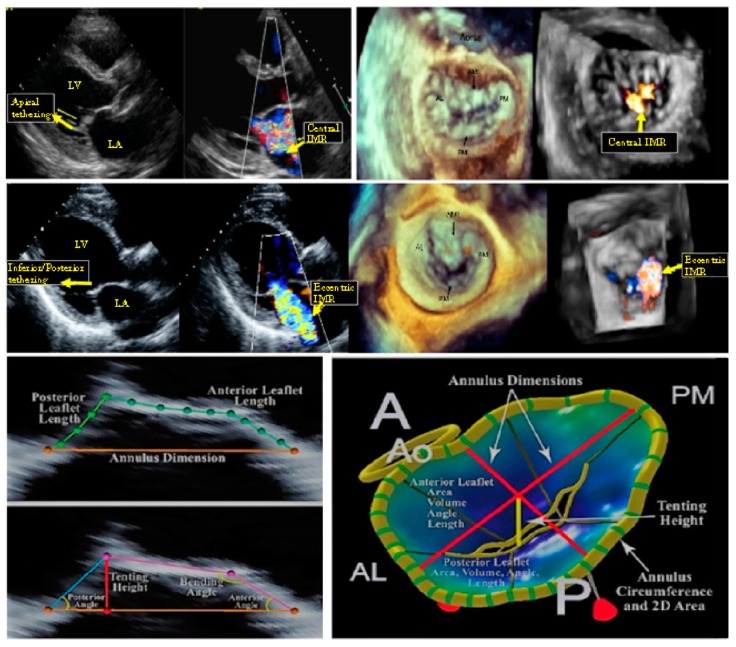
**Top Panel.** Symmetric pattern of mitral valve tethering determines biomechanical change in mitral valve configuration depicted on two- and three-dimensional echocardiography. (**Left**) Symmetric mitral valve leaflet tethering primarily in the apical direction results in a central ischemic mitral regurgitant jet. (**Right**) En face (surgeon’s view) of the mitral valve exemplifies a central, crescentic-shaped regurgitant orifice and MR jet. **Middle Panel.** Asymmetric pattern of mitral valve tethering determines biomechanical change in mitral valve configuration depicted on two- and three-dimensional echocardiography. (**Left**) Asymmetric mitral valve leaflet tethering in the inferior/posterior direction (yellow arrow) results in posteriorly-directed eccentric ischemic mitral regurgitation (IMR). (**Right**) En face (surgeons view) of the mitral valve exemplifies the resultant regurgitant orifice, which is more medially located, and the eccentric MR. **Bottom panel.** Two- and three-dimensional reconstruction of biomechanical change in mitral valve configuration. (**Left**) Representation of tenting area and tenting height. (**Right**) 3D TEE (transthoracic echography) reconstruction. AL = anterolateral commissure; AML = anterior mitral leaflet; IMR = ischemic mitral regurgitation; LA = left atrium; LV = left ventricle; PM = posteromedial commissure; PML = posterior mitral leaflet.

Echocardiographic assessment of IMR plays an important role in evaluating the mitral regurgitation severity through the direct measurement of specific geometrical parameters indicating poor LV remodelling, which has been directly associated to the grade of the pathology. Specifically, different morphological unfavourable characteristics, obtainable through diagnostic tools such as 2D echography with 3D reconstruction 4 and cardiac MRI, can independently permit the IMR classification [20,21,22]. (Figure 4)

The parameters by which the clinical practice mainly relies to assess the MV remodelling and IMR are the mitral valve diameter (MVD), the tenting area (TA) and the tenting height (TH), the anterior and posterior leaflet angles—δ_AML_ and δ_PML_, respectively—as well as the effective regurgitant orifice Area (EROA, or equivalently the vena contracta width) [7,23]. To establish LV deformation on the other hand, imaging-based diagnoses make use of IPD, along with LV end-diastolic and end-systolic diameters (LVEDD and LVESD) [21]. The different trials present in the literature have established specific ranges and threshold values of these quantities to discriminate the different states of IMR, on the basis of which the decision between valve repair and valve substitution is usually performed. For this reason, the possibility of predicting the postoperative configuration by looking at the preoperative valvular apparatus can effectively support the surgical decision-making process, by analysing the feasibility of the surgical intervention and by additionally associating the anatomical remodelling to an innovative biomechanical evaluation of the stress that the valve and the chordae experience. Assessing the internal mechanical status can indeed indicate how LV remodelling, apical displacement of ventricular wall and increased tethering forces stress the valvular and subvalvular elements in the different possible configurations. This, in turn, can provide insights into possible adverse remodelling or rupture-induced failure phenomena, permitting proper evaluation on whether or not the anatomic repair may actually lead to the effective restoration of both homeostatic loading conditions and physiological functions. By focusing on the preoperative phase, experimental measurements (see Table 1) have been interrogated to identify the initial structural deformation. For this purpose, the model uses both the end-point and the chorda preoperative inclinations for the two leaflet structures, whose joints were initially at a distance equal to MAD_pre_. For convenience, $a_{pre}^{i}$ and $b_{pre}^{i}=\left\{x_{l,pre}^{i},y_{l,pre}^{i}\right\}$, respectively, are the positions of the two joints and of the leaflet endpoints. These coordinates respect preliminary congruence with the observed preoperative leaflet angles, end-diastolic IPD and TH [4]. In particular, the requested compatibility conditions to fix the preoperative parameters are:

(11) $$y_{l,pre}^{i}=x_{l,pre}^{i}\tan\delta_{pre}^{i},$$

(12) $$TH_{pre}-\Delta TH_{pre}\leq y_{l,pre}^{i}\leq TH_{pre}+\Delta TH_{pre},$$

(13) $$IPD_{pre}-\Delta IPD_{pre}\leq d_{pre}\leq IPD_{pre}+\Delta IPD_{pre}$$

where *i* = {*a,p*}, the preoperative coordinates $y_{l,pre}^{i}$ and $x_{l,pre}^{i}$ are provided by using (6) and (7) $\delta_{pre}^{i}$ are the preoperative leaflets angles, while *d_pre_* is the interpapillary muscle distance. Denoting the preoperative location of the PMs with **m***^i^_pre_*, their distance *d_pre_* reads:

(14) $$m_{pre}^{a}=\left\{x_{l,pre}^{a}+\left(h_{pre}^{a}-y_{l,pre}^{a}\right)\tan\alpha_{pre}^{a},\;\;\;h_{pre}^{a}\right\}$$

(15) $$m_{pre}^{p}=\left\{MAD_{pre}-x_{l,pre}^{p}-\left(h_{pre}^{p}-y_{l,pre}^{p}\right)\tan\alpha_{pre}^{p},h_{pre}^{p}\right\}$$

(16) $$d_{pre}=|m_{pre}^{a}-m_{pre}^{p}|$$

**Figure 5:**
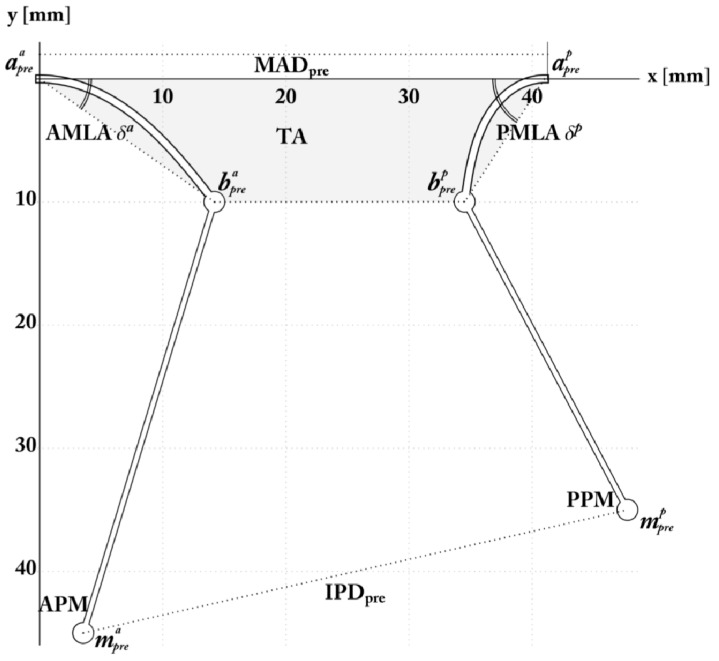
Preoperative configuration determined from the clinical dataset, with the illustration of the considered geometric factors and markers characterizing IMR grade.

The constants $h_{pre}^{a}$ and $h_{pre}^{p}$ being the annulus/papillary heads anterior and posterior heights, respectively. To precisely find the preoperative descriptors, random values of $\alpha_{pre}^{a}$ and $\alpha_{pre}^{p}$ were iteratively assigned and the congruence Equation (11) applied to numerically find the related $\theta_{l,pre}^{a}$ and $\theta_{l,pre}^{p}$, until the compatibility conditions (12) and (13) were satisfied. The preoperative parameters in Table 1 refer to average values derived from the cohort of patients reported in Nappi et al. [4], from which the preoperative configuration recalled in Figure 5 has been determined. Additionally, preoperative geometry allows calculation of the preoperative load $P_{pre}$ by using Equation (5), which is crucial to find the preoperative stretch $\lambda_{pre}^{i}$ of the chordae together with the associated reference lengths $L_{c}^{i}$ through the equilibrium (10) at the MV free edge-*chorda* nodes. One has:

(17) $$L_{c}^{i}=\frac{l_{c,pre}}{\lambda_{pre}}=\frac{\left|b_{pre}^{i}-m_{pre}^{i}\right|}{\lambda_{pre}}$$

### 2.4. Simulation and Optimization of RA and PMA

To model the reconfiguration of the preoperative apparatus via restrictive annuloplasty combined with PMA, the unknown postoperative parameters $\alpha_{po}^{i}$ and $\theta_{l,po}^{i}$ involved in the problem were associated to the aforementioned additional variables that are related to the applied displacements during the surgical intervention, i.e. the mitral annulus restriction vector r={r,0} (here applied to the posterior MV annulus, for simplicity), and the PM displacement vectors **u***^a^* and **u***^p^*. In terms of their components, these vectors were respectively written as $u^{a}=\left\{u_{x}^{a},\;\;\;\zeta^{a}u_{x}^{a}\right\}$ and $u^{p}=\left\{-u_{x}^{p},\;\;\zeta^{p}u_{x}^{p}\right\}$, the coefficients *ζ^a^* and *ζ^p^* indicating the orientation of each papillary displacement towards the predicted postoperative position. Then, the nodes of the MV structure can be redefined in the current configuration in terms of the nine unknowns just described. One finds the posterior MV joint in position $a_{po}^{p}=a_{pre}^{p}-r$, the leaflet edges respectively in position $b_{po}^{a}=\left\{x_{l,po}^{a}(\theta_{l,po}^{a},\alpha_{po}^{a}),\;y_{l,po}^{a}(\theta_{l,po}^{a},\alpha_{po}^{a})\right\}$ and $b_{po}^{p}=\left\{x_{l,po}^{p}(\theta_{l,po}^{p},\alpha_{po}^{p}),\;y_{l,po}^{p}(\theta_{l,po}^{p},\alpha_{po}^{p})\right\}$, defined by means of the Equations (6) and (7), while the papillary muscles move to $m_{po}^{a}=m_{pre}^{a}+u^{a}$ and $m_{po}^{p}=m_{pre}^{p}+u^{p}$ in greater detail. Suitable relations are provided in order to identify the postoperative configuration. In particular, equilibrium (10) is imposed at both the free nodes:

(18) $$\sigma_{po}^{i}=\lambda_{po}^{i}\frac{P_{po}}{A_{ref}}$$

in which $\lambda_{po}^{i}=|b_{po}^{i}-m_{po}^{i}|/L_{c}^{i}$ is the total stretch, the chord stress $\sigma_{po}^{i}$ coming from the Equation (9) by setting $2\epsilon ={\left(\lambda_{po}^{i}\right)}^{2}-1$ while the leaflet current load $P_{po}^{i}=P^{i}(\theta_{l,po}^{p},\;\alpha_{po}^{p})$ is evaluated by using (5). Equilibrium Equations (18) are accompanied by two congruency conditions that relate the Lagrangian parameters $\alpha_{po}^{i}$ to the *chordae* orientation, in other words:

(19) $$\begin{array}{l} \frac{\pi }{2}+\alpha_{po}^{a}=\arg\left(m_{po}^{a}-b_{po}^{a}\right)\\ \frac{\pi }{2}-\alpha_{po}^{p}=\arg\left(m_{po}^{p}-b_{po}^{p}\right) \end{array}$$

Furthermore, problem-specific constraints were introduced to ensure both mitral leaflets meet at a desired coaptation height, say *TH^opt^*, and that the mitral annulus antero-posterior diameter reduces by *r*, in other words:

(20) $$\begin{array}{l} y_{l,po}^{i}=TH^{opt},\;\\ x_{l,po}^{a}+x_{l,po}^{p}=MAD_{pre}-r \end{array}$$

**Figure 6:**
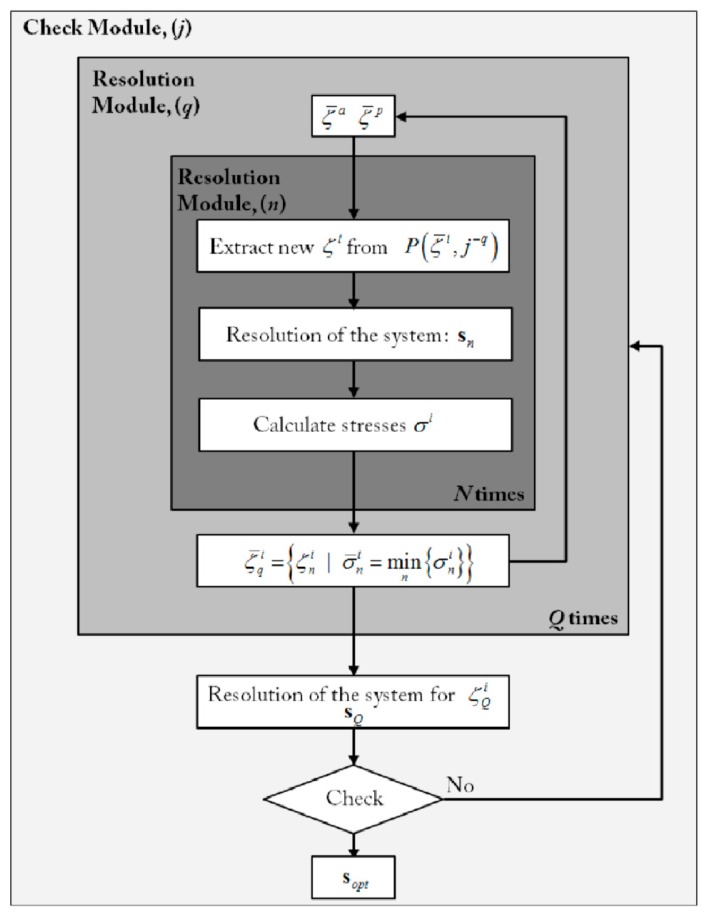
Conceptual scheme of the solution strategy. Starting from random search, the postoperative configuration is determined by adopting a minimum-stress criterion.

In this way, relationships (18), (19) and (20) form a system of seven equations. To find the most suitable current configuration, the orientation of the PM displacement vectors ζ*^a^* and ζ*^p^* were treated as additional design variables, determined to optimize the PMA in such a way to minimize the chordae tendinea stress. This was achieved by implementing an ad hoc random iterative procedure, schematized in Figure 6.

## 3. Results

### 3.1. Mitral Valve: Annulus and Leaflet

**Figure 7:**
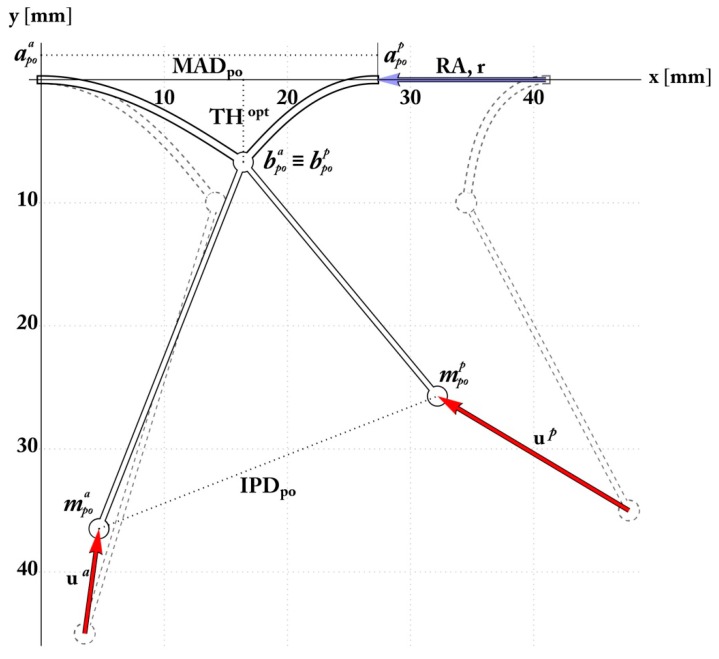
Analytically determined postoperative configuration showing mitral valve (MV) restriction, leaflet coaptation, and optimal interpapillary muscle distance (IPMD) ensuring the minimum stress within the tethering chordae.

**Figure 8:**
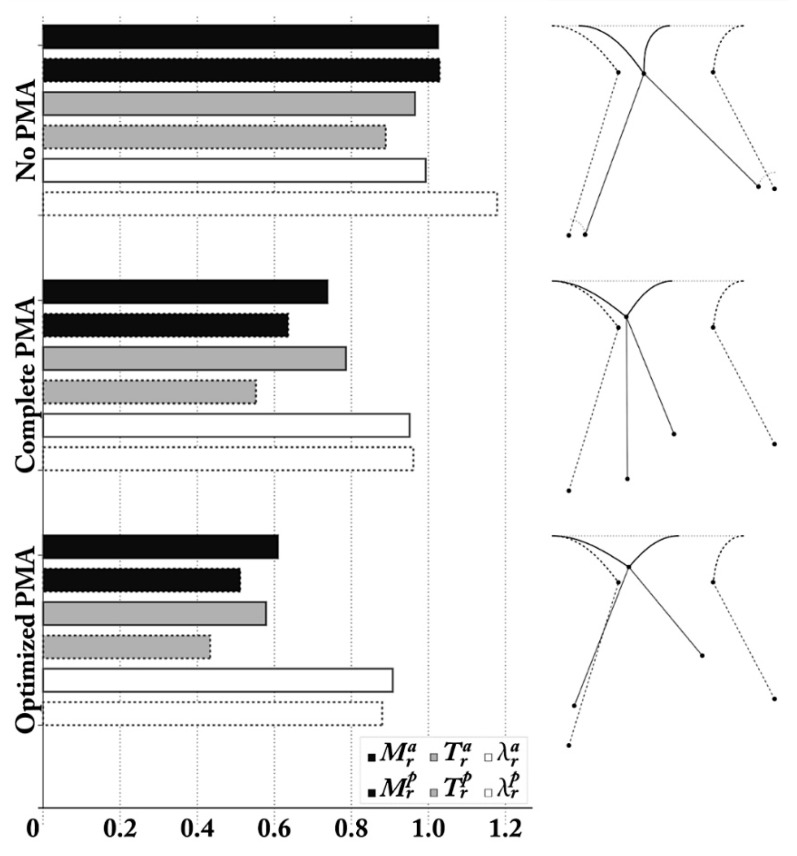
Comparison of the optimized postoperative outcome with both absent PMA and complete PMA. Different outcomes were obtained by starting from the same pre-operative configuration. In each case, effectiveness of the outcomes was measured by means of non-dimensional indicators in terms of *i*) valvular annulus stress ratio (black bars), measured as the ratio between post-operative and pre-operative bending moments predicted in the leaflets roots $M_{r}^{i}=M_{po}^{i}/M_{pre}^{i}$. *ii*) *Chordae* stress ratios $T_{r}^{i}=T_{po}^{i}/T_{pre}^{i}$, evaluated as the ratio between the post-operative and the pre-operative nominal axial stresses of the *chordae tendinae* (Gray bars). *iii*) Stretch ratios of the *chordae tendinae* $\lambda_{r}^{i}=\lambda_{po}^{i}/\lambda_{pre}^{i}$ (white bars), denoting the ratio between post-operative and pre-operative lengths of the chordae. Bars with dashed contours refer to posterior leaflets.

The postoperative configuration is shown in Figure 7 and refers to an end-systolic configuration with valve closure. The comparison between the theoretically predicted parameters and the effective postoperative ones are reported in Table 2. From these data, the minimum restriction r (w.r.t. the adopted parameter) required to obtain leaflet coaptation was about 14 mm, thereby t reduced the MAD (Mitral Anular Diameter) from 41 to 26.9 mm, which is in full agreement with the postoperative follow-up by Nappi et al. [4,5,6]. Furthermore, valve re-shaping and restored coaptation allowed attenuation of leaflet tethering and reduced the mechanical stress experienced by the MV annulus, at the anterior and posterior hinge points. The MA stress was correlated to the reactive bending moments, which theoretically diminished by 40% and 50% in the anterior and posterior annulus, respectively, according to Figure 8.

### 3.2. Mitral Valve: Subvalvular Apparatus

The theoretical predictions also highlight the optimal positioning of the PMs, achieving an end-systolic IPD of about 27.5 mm, actually suggesting a 28% reduction of the ED-IPD (End- diastolic-interpapillary muscle distance), in accordance with the clinical PMA cases. Additionally, the mathematical model indicates the optimal subvalvular arrangement and the predicted PM positions in agreement with the displacement executed in surgery, as reported in Table 2. The PMs postoperative locations are identifiable through the PM head-annular distance and IPD. From a mechanical standpoint, the chordae elastic stretches as the axial stress decreases, this being relevant to avert the risk of a yielding phenomenon in the chordae tendineae that would compromise their functionality.

### 3.3. Combined Valvular and Subvalvular Model

To account for the final hindering aspect, the model was implemented to compare the effects of RA combined with PMA. Additional scenarios were simulated, by considering the sole RA and the biomechanics-guided PMA. Analyses highlighted the capability of this strategy to beforehand recognize—at least theoretically—the leading biomechanical factors governing the expected outcome, thus envisaging the use of biomechanics for orienting surgeons’ choices and improve PMA in IMR treatment. By prescribing coaptation, the main differences indeed occurred in the subvalvular apparatus. In RA alone, papillary muscles were subjected to a small prescribed displacement located in the end-systolic position with no additional approximation unlike the optimized case. The valve treatment was entirely imputed to annulus restriction assuming the PMs are motionless. However, in this case, the simulation of the sole RA still provided equilibrium alongside congruence Equations (19) and (20), while coaptation constraints (21) were slightly modified by decoupling the restriction *r* into two variables (i.e., $r=r^{a}+r^{p}$) to consider the restriction with respect to each PM. Next, in the complete PMA, the PMA displacement orientation was fixed, and an additional equation was introduced prescribing the postoperative IPD (equal to 10 mm, to match some clinical evidence in the literature [24]. Starting from the same preoperative conditions, results were compared with the optimized postoperative situation by means of non-dimensional effectiveness indicators:

Valve anulus stress ratio, estimated as the ratio between the post-operative and pre-operative bending moments of the anterior and posterior elasticas predicted by the analytical model in correspondence of the valve roots, given by $M_{r}^{i}=M_{po}^{i}/M_{pre}^{i}$

*Chordae* stress ratio $T_{r}^{i}=T_{po}^{i}/T_{pre}^{i}$ measuring the eventual stress relaxation of tethering forces in chordae tendinae when passing from pre-operative to post-operative configurations. 

*Chordae* stretch ratio $\lambda_{r}^{i}=\lambda_{po}^{i}/\lambda_{pre}^{i}$, which evaluates ratio between the post-operative and pre-operative lengths of the chordae tendinae.

As shown in Figure 8, the absence of PMA produces increased *chordae tendineae* stretching and tethering forces with respect to the preoperative condition. This potentially compromises the mechanical seal of the leaflet coaptation and may be a prodromal signal to secondary reopening.

## 4. Discussion

Mitral repair for IMR is currently undergoing a paradigmatic shift in stating the need to address the mitral valve apparatus in its entirety (i.e., annulus, leaflet and subvalvular apparatus). The current clinical evidence suggests positive results with PM surgery [4,5,6,25,26], but the type and the extent of repair as well as the degree of residual tethering after surgery represents the Achilles’ heel of this surgery. Despite some echocardiographic predictors [27], the evaluation of MR reduction and the possibility of predicting the success of both the related surgical procedures and the response to treatment are all still ongoing issues [22]. In this framework, we propose a biomechanical model to support the heart team decision making process in mitral annuloplasty with combined PMA. The major findings of this study are: 1) Simulations focus on preoperative symmetric tethering of types III-IV and by setting a target coaptation distance *TH_opt_*, in line with physiological values of tenting heights from healthy control groups and successful follow-ups, reported in Table 1 (however, the model can evenly reproduce asymmetric patterns). 2) Theoretical outcomes, derived through the procedure of Section 3, provided the magnitude of the annular restriction, suggesting an estimation of the MA ring diameter for the intervention. 3) Contemporaneously, we obtained the optimal IPD potentially achievable through PMA with e-PTFE (expanded Polytetrafluoroethylene) material in order to restore the most favourable homeostatic stress conditions. Concerns regarding homogeneous distribution of stress on dysfunctional papillary muscle due to regional myocardium infarction and ischemia were resolved by the addition of e-PTFE. Previously we performed the repositioning of the PMs by using autologous pericardium or Teflon, which were not suitably compliant to both shear modulus requirements and the different levels of systolic-diastolic stress of the cardiac cycle. Indeed, ePFTE is a material that has specific elastomechanical properties, known as auxetic behaviour for its negative Poisson’s ratio. Thus, the material’s ability to respond to applied forces provides extremely advantageous compliance properties. When subjected to a tensile stress, ePTFE fibres open up structurally and expand tangentially to the stress; conversely, if these materials are subjected to compression, they close structurally [28,29,30,31,32]. In our study, we observed that, although complete PMA relaxes valve tethering and valve root strain, stress and stretch ratios were significantly greater than in the biomechanically-driven optimized PMA case (Figure 8), thus demonstrating the advantage of predicting the effective postoperative distance at which PMs should be placed. Additionally, the model highlights that a severe-sized PMA implies that disproportionate displacements *u^a^* and *u^p^* were prescribed, leading to the increase of higher drag forces within both PM roots and ventricular walls: consequently, an over-estimated correction could potentially compromise the local ventricular wall motility and functionality, as well as the stability of the subvalvular implant because of the pull of the muscle wall reaction forces. 

We propose a simple structural scheme, in which nonlinear elastic solutions are founded by incorporating all the key geometrical and mechanical factors governing the problem and are actually considered in the preoperative, operative and post-operative phases. Our results confirm the leading variables considered in the surgical procedure, including the pivotal role of the direction of PM displacement. The model highlighted how the morphological factors are directly involved in the estimation of postoperative outcomes, revealing how the whole process and the postoperative outcome depend on the synergistic action of initial geometry, deformed configurations and in situ evolving mechanical stresses. Preoperative evaluation of chordae and annular stresses can be extremely helpful in understanding the physical status of the valvular and subvalvular systems and to know how the severity of IMR compromises the health of the structure. An excessively stressed structure can in fact undergo local yielding phenomena, which in turn can compromise the elastic properties of the apparatus and, consequently, its functionality. It is felt that the proposed *Elastica*–based biomechanical model, by integrating geometrical data and mechanical stresses, could advantageously represent a complementary tool to orient the surgeon’s choices in PMA procedures, and provide a first step towards the definition of a biomechanical index able to predict effectiveness of MV repair, suggest optimal design protocols for LV surgical remodelling and plan PMA success.

## 5. Limitation and Perspective

Authors acknowledge several limitations in this study. Firstly, the model was based on echocardiographic measure of TH, IPMD, mitral annulus diameter and LV remodelling and no 3D reconstructions were performed. This would have required the inclusion of normalization parameters of the mitral valve geometry following the surgical management of symmetric or asymmetric deformations. Indeed, the model introduces some simplifying hypotheses, the most important of which being the two-dimensional kinematics, which; thus, sacrifices more realistic representations of the MV, obtained for example by resorting to approaches based on finite elements [24,28]. In fact, 3D models allow consideration of more faithful geometries of the MV, by considering membrane/shell structures to represent valve leaflets (hence including the effects of the curvature normal to the beam plane) and heterogeneous properties [24], as well as analysing the effects of the systolic pressure on the valve deflection. Provided the general relevance of all these aspects for the analysis of in situ healthy MV, it is worth to highlight that the present approach aims to simulate PMA during the intervention; therefore, in the absence of the effects of systolic pressure on the leaflets. Moreover, the in-plane projection of the MV well traces its essential movements, as also experimentally observed [16], even while undergoing PMA. Its most significant geometrical parameters derive from 2D echographic observations, used both in assessing the IMR severity [27] and in selecting the appropriate treatment strategy (repair vs. replacement). It is felt that some three-dimensional features inevitably lost in the present model are compensated by the parametric form of the equations—and of the associated solutions—obtained in the proposed mathematical formulation. The formulas in fact furnish a versatile tool to support surgeons for planning the best practice, in real-time visualizing essential stress and geometrical conditions, which would occur after the intervention and that 3D Finite Element simulations would only determine with significantly higher computational costs and time-consuming analyses. Finally, the model was designed to determine solid basic measurements to analyse and provide surgical input in conditions of asymmetric and symmetric tethering. Additional studies should be performed with the aid of 3D reconstructions to evaluate other forms of mitral geometric abnormalities.

## Figures and Tables

**Table 1:** Preoperative geometrical measurements and constitutive parameters, adapted from [4].

**Preoperative Measurement** **Name^source^ and Unit**	**Value (mean ± std)**
Mitral Annulus Diameter *AD_pre_*, mm End-Diastolic Interpapillary	40 ± 1.9
Muscle Distance *ED IPMD*, mm	44.6 ± 0.39
Tenting Height *TH^pre^*, mm	12.4 ± 0.13
Target Coaptation Distance *TH^opt^*, mm	6.8
Anterior Mitral Leaflet Angle *δ^a^* [°]	32.6° ± 2.5°
Posterior Mitral Leaflet Angle *δ^p^* [°]	56.8° ± 5.2°
Anterior Annulus to Papillary Head *h^a^*, mm	45 ± 8.6
Posterior Annulus to Papillary Head *h^p^*, mm	28 ± 8.1
**Model Parameters** **Name^source^ and Unit**	**Value**
Anterior leaflet length^31^ *l^a^,* mm	18
Posterior leaflet length^31^ *l^p^,* mm	13
Leaflet bending stiffness^10^ *B*, mN mm^2^	500
Chordae tendineae material constants^31^ *c*1, mN mm^−2^ *c*_2_, [-]	352.4 0.1907
Chordae tendineae nominal area^32^ *Aref*, mm^2^	0.197

**Table 2:** Comparison between analytical predictions and surgical outcomes.

Postoperative Configuration	Analytical Prediction (Preoperative Value)	Surgical Outcome (mean ± std)
*MAD*, mm	26.9(41)	24.6 ± 2.4
*ES IPMD*, mm	27.5	32.7 ± 3.2
*h^a^*, mm	38(45)	37.5 ± 8.2
*h^p^*, mm	26(35)	23 ± 7.4

MAD: ES IPMD: end systolic interpapillary muscle distance.

## Abbreviations

MV	Mitral Valve
LV	Left Ventricle
RA	Restrictive Annuloplasty
PMA	Papillary Muscle Approximation
PM(s)	Papillary Muscle(s)
IPD or IPMD	Interpapillary Muscle Distance
ES, ED	End Systolic, End Diastolic
MAD	Mitral Annulus Diameter
PMPM	Posteromedial Muscle
ALPM	Anterolateral Papillary Muscle
TH	Tenting Height
LVESD	Left Ventricular End Systolic Diameter
LVEDD	Left Ventricular End Diastolic Diameter
EROA	Effective Regurgitant Orifice Area
ACC	American College of Cardiology
AHA	American Heart Association
